# 

**Published:** 1896-04-18

**Authors:** 


					THEIIHOSPITAL. ABSOLUTELY PURE, therefore BEST* Apbil 18th, 1896.
CADBURY'S ww ^ CADBURY'S
COOOA nr IT? IT1 COCOA
"The standard of highest purity at present J|^> '?1 i NO ALKALIES UESD.,
attainable,"?Latutt,
R W1CH HOSfi.;
No. 49 ?. Vol. XX.
Saturday,
April 18th, 1896.
WITH EXTRA NUR8INB SUPPLEMENT. Pp^?L.OT?5S?b*
[Registered at the General Post Offlce as a Newspaper tor Monthly Parts, 6d.; post free, Ed
transmission in the United Kingdom and Abroad.] Ditto per Annnm.8s.bd., post fref.

				

